# A multicentre phase III randomised controlled single-masked clinical trial evaluating the *cl*inical *e*fficacy and safety *o*f light-masks at *p*reventing dark-*a*daptation in the *tr*eatment of e*a*rly diabetic macular oedema (*CLEOPATRA*): study protocol for a randomised controlled trial

**DOI:** 10.1186/1745-6215-15-458

**Published:** 2014-11-22

**Authors:** Sobha Sivaprasad, Geoffrey Arden, A Toby Prevost, Roxanne Crosby-Nwaobi, Helen Holmes, Joanna Kelly, Caroline Murphy, Gary Rubin, Joanna Vasconcelos, Philip Hykin

**Affiliations:** NIHR Moorfields Biomedical Research Centre, 162, City Road, London, EC1V 2PD England; City University, London, England; KCL Department of Primary Care and Public Health Sciences, NIHR Biomedical Research Centre at Guy’s and St Thomas’ NHS Foundation Trust and King’s College, London, England; King’s Clinical Trials Unit at KHP King’s College London, London, England; Institute of Ophthalmology, University College London, London, England

## Abstract

**Background:**

This study will evaluate hypoxia, as a novel concept in the pathogenesis of diabetic macular oedema (DMO). As the oxygen demand of the eye is maximum during dark-adaptation, we hypothesize that wearing light-masks during sleep will cause regression and prevent the development and progression of DMO. The study protocol comprises both an efficacy and mechanistic evaluation to test this hypothesis.

**Method/Design:**

This is a phase III randomised controlled single-masked multicentre clinical trial to test the clinical efficacy of light-masks at preventing dark-adaptation in the treatment of non-central DMO. Three hundred patients with non-centre-involving DMO in at least one eye will be randomised 1:1 to light-masks and control masks (with no light) to be used during sleep at night for a period of 24 months. The primary outcome is regression of non-central oedema by assessing change in the zone of maximal retinal thickness at baseline on optical coherence tomography (SD-OCT). Secondary outcomes will evaluate the prevention of development and progression of DMO by assessing changes in retinal thickness in different regions of the macula, macular volume, refracted visual acuity and level of retinopathy. Safety parameters will include sleep disturbance. Adverse events and measures of compliance will be assessed over 24 months. Participants recruited to the mechanistic sub-study will have additional retinal oximetry, multifocal electroretinography (ERG) and microperimetry to evaluate the role of hypoxia by assessing and comparing changes induced by supplemental oxygen and the light-masks at 12 months.

**Discussion:**

The outcomes of this study will provide insight into the pathogenesis of DMO and provide evidence on whether a simple, non-invasive device in the form of a light-mask can help prevent the progression to centre-involving DMO and visual impairment in people with diabetes.

## Background

Diabetic retinopathy is the most common complication of diabetes. Diabetic macular oedema (DMO), characterised by leakage of fluid from compromised blood vessels in the central retina, is the most frequent cause of visual impairment in people with diabetes. DMO may be central or non-central oedema. Non-central oedema does not usually affect visual acuity. When it affects the central 1 mm of the macula, it causes visual impairment. Over 30% of eyes with untreated centre-involving macular oedema lose 3 or more lines of vision by 5 years [1].

Patients with non-central DMO are monitored with slit-lamp biomicroscopy and spectral domain optical coherence tomography (SD-OCT) every 4 to 6 months for progression to centre-involving DMO. SD-OCT provides information on the changes in the retinal thickness and morphology of the retina due to DMO. Approximately 30% of these patients progress to centre-involving macular oedema by 12 months [2].

Treatment is available only when the DMO becomes clinically significant or shows progression to the centre. Laser treatment is the standard of care when the DMO becomes clinically significant. Although laser treatment reduces the risk of moderate visual loss by 50% at this stage, it is not effective in restoring visual acuity and has significant side effects that impact on the quality of life of these people [1]. Newer treatment options of injections of vascular endothelial growth factor (VEGF) inhibitors are also available but only for centre-involving DMO. These treatments are costly and cause significant burden to the patient, their caregivers and the healthcare system [3, 4].

There are no treatment options for non-clinically significant DMO except optimal control of diabetes and hypertension. Laser photocoagulation may be performed for non-central clinically significant macular oedema. The natural history of the disease is to progress from non-central to centre-involving DMO [2]. Therefore, there is a substantial unmet need for both treatment and prevention of progression of non-centre-involving DMO.

The exact pathogenesis of diabetic retinopathy and DMO is uncertain. The rationale for this study is that increased glucose is associated in various ways with a decrease in oxygen supply to the retina, and an increase in oxygen demand [5]. This leads to increased hypoxia, and an overproduction of VEGF, which damages the circulation, and in doing so will further decrease retinal oxygen supply in a vicious circle. Only at such a stage will all the other known mechanisms that contribute to retinal vascular damage operate and contribute to the various clinical features of diabetic retinopathy. Rods use more oxygen than any other cell in the body [5, 6]. Oxygen is required to support the extreme sensitivity to light that develops during dark-adaptation. As a result, the oxygen tension in the mitochondrial region of the rods in darkness falls to zero. The exact mechanism is that in darkness the rod outer segment membrane becomes extremely permeable to ions and water, which enter the cell and are pumped out in the inner segment [7]. The resulting ‘dark current’ is large and requires all the oxygen available in the normal eye [8–10]. If retinal circulation is compromised in any way, the hypoxia present in the outer retina increases and spreads into the inner part.

Arden hypothesized that if dark-adaptation was prevented, the rod dark current would never become maximal and diabetic retinopathy would be alleviated by decreasing the oxygen demand [8, 9]. Since people only dark-adapt at night during sleep, sleeping in an illuminated environment should prevent or reverse the condition. In clinical trials it is important to provide uniform illumination to each patient, so we have made ‘light-masks’ containing organic light emitting diodes to illuminate the closed eyelids during sleep. Sufficient low intensity light is transmitted by the lids to reduce the dark current allowing the quantity of light can be measured. It is also important that the masks are comfortable to wear and do not disturb sleep.

The relationship of these SD-OCT changes in retinal morphology and thickness to hypoxia has not been determined. Similarly, the relation of the visual function to hypoxia also remains unclear. This is the subject of the mechanistic investigation.

### Objectives

The primary objective of the study is to explore whether wearing light-masks during sleep at night reduces, relative to the control masks, the maximal zone macular thickness at baseline as measured by SD-OCT in the study eye of patients with non-centre-involving DMO at 24 months.

Secondary objectives include whether the effect of light-masks, relative to control masks, can prevent the progression of non-centre-involving DMO by assessing the changes in retinal thickness in each region of the macula, macular volume, macular morphology, retinopathy status, visual acuity and proportion of patients requiring rescue laser or anti-VEGF treatment at 12 and 24 months. The safety and tolerability of the masks will be assessed and will include measures of compliance and questionnaires to assess the effect of the masks on sleep.

The mechanistic evaluation will explore and compare the changes in retinal function induced by supplemental oxygen and light-mask on multifocal ERG (inner retina) and scotopic microperimetry (outer retina) at 12 months. The changes in oxygen saturation in the retinal vessels will be assessed by retinal oximetry using the Oxymap T1 Retinal Oximeter (Oxymap Analyzer v.2.4.2, Reykjavik, Iceland

## Methods/Design

**Figure 1:**
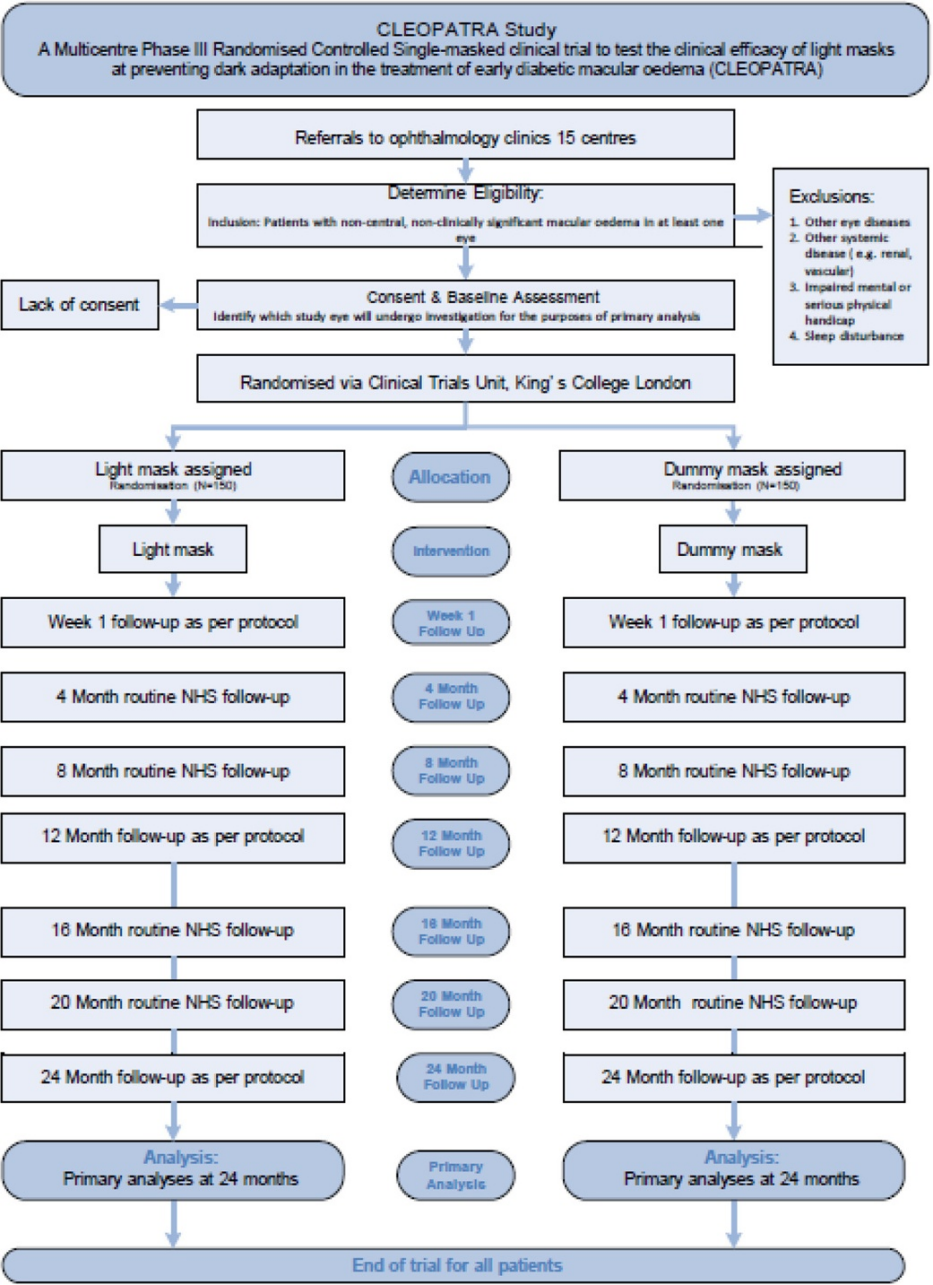
**Flow diagram of the CLEOPATRA trial.** The flow diagram shows the patient flow through the period of the trial and includes the mechanistic evaluation done at one site only.

This is a phase III randomised controlled single-masked clinical trial that will evaluate the efficacy and safety of light-masks in treating and preventing the progression of non-centre-involving DMO. Three hundred patients with non-centre-involving DMO in at least one eye will be randomised 1:1 to light-masks and control masks (with no light) to be used during sleep at night for a period of 24 months. This basic study design and the associated clinical measurements are well-established, having been successfully used in numerous previous clinical trials of DMO. These include visual acuity and retinal thickness measurements using SD-OCT every 4 months, refracted visual acuity, retinal colour photographs, blood pressure, glycosylated haemoglobin (HbA1c), Pittsburgh Insomnia Rating Score [11] and Epworth Sleepiness Scale [12] questionnaires to assess sleep at baseline, 12 and 24 months, adverse events and measures of compliance at all 8 study visits over 24 months. Best corrected visual acuity will be repeated at baseline and SD-OCT assessment will be done twice at 12 and 24 months to assess inter-test variability. Participants recruited to the mechanistic evaluation will have additional retinal oximetry, multifocal ERG and microperimetry at baseline and 12 months. Please see visit schedules (Table 1) and study flow diagram (Figure 1). At least 15 sites in England and Wales will be participating in this study. Kings Clinical Trials Unit (KCTU) is the co-ordinating centre. The study has been approved by the National Research Ethics Service Committee London - Dulwich (Ref: 13**/**LO**/**0145).

**Table 1:** **CLEOPATRA - summary of study assessments**

	Baseline	Week 1	Month 4	Month 8	Month 12	Month 16	Month 20	Month 24 End of trial	
Study Week/Month	Visit 1	Visit 2	Visit 3	Visit 4	Visit 5	Visit 6	Visit 7	Visit 8	Withdrawal
Registration/Demographics	x								
Informed consent	x								
Eligibility form	x								
Randomisation form	x								
Medical history	x								
Concomitant medications	x		x	x	x	x	x	x	x
HbA1c	x				x			x	x
BP	x				x			x	x
Pittsburgh Sleep Quality Index & Epworth Sleepiness Scale	x	x			x			x	x
BCVA (refraction at baseline, 12 and 24 months)	x		x	x	x	x	x	x	x
BCVA (repeated at baseline)^a^	x								
OCT - macular thickness protocol^b^	x		x	x	x	x	x	x	x
Colour photographs - 3-field	x				x			x	x
Mask compliance form		x	x	x	x	x	x	x	x
Adverse events form		x	x	x	x	x	x	x	x
Withdrawal form									x
Additional tests for mechanistic evaluation (n = 30)									
Microperimetry dark-adapted	x				x				
mfERG	x				x				
Retinal oximetry	x				x				

BCVA: best corrected visual acuity; OCT: optical Coherence Tomography; mfERG: multifocal electroretinogram; BP: blood pressure. Note: where an ‘x’ is contained within a field this denotes that the associated data will be collected at the identified time point. ^a^denotes that visual acuity with new refraction should be repeated at baseline. ^b^denotes that OCT scans should be repeated at month 12 and 24 to test for inter-test variability. The average of the two central subfield thicknesses on OCT will be used for the analysis.

### Patient recruitment

Patients may be identified from diabetic retinopathy screening programmes and medical retina clinics of the trial sites and its satellite clinics. In addition, patients may be referred by other medical retina consultants from other hospitals to the Principal Investigators (PIs). In order to prevent patients from being subjected to unnecessary trial procedures, it is recommended that potential participants have an SD-OCT done at their clinic visit or at a pre-screening clinic before trial screening procedures are done to ensure exclusion of eyes with centre-involving macular oedema defined as central subfield > 300 μm.

We will report on the numbers of eligible patients informed of the study, the numbers screened and the numbers who were recruited into the study.

Inclusion criteria are:

- Subjects of either sex aged 18 years or over
- Diagnosis of diabetes mellitus (type 1 or type 2). Any one of the following will be considered to be sufficient evidence that diabetes is present:
- Current regular use of insulin for the treatment of diabetes
- Current regular use of oral anti-hyperglycaemic agents for the treatment of diabetes
- Documented diabetes by ADA and/or WHO criteria
- Best corrected visual acuity in the study eye better than 55 ETDRS letters (Snellen VA 6/24)
- On clinical exam, retinal thickening due to early DMO not involving the central 1000 μm of the macula characterised by presence of microaneurysm, exudates or oedema and OCT evidence of increased retinal thickness in at least 1 non-central ETDRS zone of ≥320 μm.
- Previous macular laser, intravitreal steroids or anti-VEGF treatment is permitted provided the last laser treatment was done at least 4 months before date of recruitment or anti-VEGF treatment was done at least 2 months previously
- Media clarity, pupillary dilation and subject cooperation sufficient for adequate fundus photographs
- Ability to return for study visits
- Ability to give informed consent throughout the duration of the study

Exclusion criteria include:

1. Clinical evidence of centre-involving macular oedema that requires laser treatment within the next 6 months (central subfield on OCT > 300 μm)
2. Macular oedema is considered to be due to a cause other than DMO
3. An ocular condition is present (other than diabetes) that, in the opinion of the investigator, might affect macular oedema or alter visual acuity during the course of the study (for example, vein occlusion, uveitis or other ocular inflammatory disease, neovascular glaucoma, Irvine-Gass syndrome, and so on).
4. History of treatment for DMO at any time in the past 4 months (such as focal/grid macular photocoagulation, intravitreal or peribulbar corticosteroids, anti-VEGF drugs, or any other treatment) in the study eye
5. History of panretinal scatter photocoagulation in the study eye
6. Active proliferative diabetic retinopathy in the study eye
7. A condition that, in the opinion of the investigator, would preclude participation in the study
8. Corneal scarring, vitreous opacities, severe asteroid hyalosis that would inhibit proper visualisation, inability to be positioned in front of the SD-OCT device, inability to understand the requirements of the imaging, and nystagmus
9. Patients with active insomnia or any other relevant sleep disturbances

### Consent procedure

Eligible patients are informed about the study by a member of the clinical team or research staff and written information sheet is provided. After a consideration of at least 24 hours, the patient is contacted to enquire whether the patient is willing to participate in the study. At the screening visit, a written information consent approved by the local ethics committee is obtained from all participants before any trial-related procedures are performed.

### Randomisation

Randomisation will be via a bespoke 24-hour web-based randomisation system hosted at the King’s Clinical Trials Unit (KCTU). Patients will be randomised at the level of the individual, using the method of minimisation incorporating a random element. The minimisation factors will be HbA1C (< 7.999% (63.89 mmol/mol or below) or ≥8% (69.90 mmol/mol or above), perifoveal or parafoveal location of baseline zone of increased retinal thickness of ≥320 μm (parafoveal zone 2 to 5 or perifoveal zone 6 to 9) and study site. If both parafoveal and perifoveal zone thickening co-exist, it will be categorised as parafoveal.

Patients may only be randomised into the study by an authorised member of staff at the study site as detailed on the delegation log. Participants may only be randomised into the study once.

### Trial interventions

#### Light-masks

The light-mask is manufactured by PolyPhotonix Medical Ltd., Petec Netpark, Sedgfield, TS21 3FG, UK and will be purchased from this company and supplied directly to trial sites.

The light-mask is a device designed to deliver a precise phototherapy to a user’s retina through closed eyelids. The light-mask comes in two parts, a fabric mask and a light emitting unit, or ‘Pod’. When worn, the Pod is inserted into the fabric mask and placed over the patient’s eyes and attached using an adjustable Velcro strap. The Pod contains two Organic Light Emitting Diodes (OLEDs), which will be located over the eyes of the patent when the light-mask is being worn. The fabric mask is made of nylon, polyurethane and polyester. These materials are non-toxic and are commonly used in a wide variety of skin-contacting apparel. The Pod is made from medical grade low-density polyethylene, which has been tested and passed the relevant physiochemical and *in vivo* biological reactivity tests required for the USP < 88 > Class VI requirements. The Pod and fabric mask have been designed to be thin and flexible and contoured to compliment the face and improve comfort for the wearer.

The OLEDs are powered by two 3 V (CR2450) batteries which power the device without the need for an external power source or recharging. At the end of the mask’s lifetime a replacement light-mask is required. A new fabric mask will be provided with each mask to minimise contamination resulting from continued use. The mask is time, date and touch sensitive. The mask will only ‘work’ between pre-determined operational windows - typically 8 pm to 10 am during the light-mask’s lifetime. Within these times the mask can be activated by a light touch. If worn within 3 minutes of activation, sensors on the Pod will keep the mask illuminated for the night’s therapy. The times for which the mask is worn will be logged for compliance analysis. The light-mask has CE certification as a Class 2a device and its design and manufacture meet the standards of ISO13485.

The design must permit the mask to be worn by people with different head shapes and deliver rod excitation efficiently. The spectral output is important and should be matched as closely as possible to the response spectrum of the rod cells. This has been tested in two clinical trials. The first was a proof of concept study, in which 12 patients slept in a mask containing a chemoluminescent source which exposed one eye only to light. The trial lasted 3 months. All found the masks comfortable and the method of treatment acceptable. There were no reports of adverse effects. Measurements of colour contrast sensitivity and examination of standard fundus photographs showed that in the ten for whom complete records were available, colour vision improved and the area of retina covered by microaneurysms and small dot haemorrhages decreased. These results were significant even though the trial was very short and the numbers treated were so few [13].

A second study was carried out using electronic sources of light - blue-green light emitting diodes (LEDs) to illuminate one eye. The electrical power of the system was < 3 mW. Forty patients were recruited and follow-up visits were at 3 and 6 months. All patients had early DMO in one eye and the other eye was used as control. A total of 34 (85%) out of 40 patients completed the study. Twenty-eight study eyes showed intraretinal cysts compared with nine in the fellow eyes. At 6 months, only 19 study eyes had cysts while cysts were seen in 20 fellow eyes. The zone of maximum thickness showed a reduction of retinal thickness by 12 μm (95% CI 3 to 21, *P* = 0.01). The secondary outcomes of change in visual acuity, achromatic contrast sensitivity, and microperimetric thresholds improved significantly in study eyes and deteriorated in fellow eyes [14].

The patients in this study will wear the light-mask each night, receiving a maximum of 8 hours therapy per night. The optical output of the mask has been tuned to optimise scotopic intensity while minimising photopic intensity. The masks regulate the light output to a constant luminosity x:


This is well below toxic levels of luminosity but of sufficient scotopic intensity to prevent dark-adaption. Emission below 470 nm is less than 3% of total output posing little or no risk of harm.

Every mask is capable of recording precisely when and for how long it has been used, thus providing a very accurate measure of compliance. Each mask will have a predetermined lifetime and will need returning and replacing when this time expires. On the return of each mask the compliance data can be downloaded and analysed. Patients will receive phone reminders and/or counselling.

#### Control masks

The control masks will consist of the CE marked device with all internal electronics and functionality removed and it is worn in the same way as the CE marked device.

### Blinding

Control participants will be provided with identical dummy masks with no active light. However, it is likely that patients in the control arm may not wear these masks without illumination for a period of two years as they are not masked to treatment allocation but all efforts will be taken to ensure compliance. Primary outcome assessors (optometrists and SD-OCT technicians) will remain masked to treatment allocation. The optometrists are the visual acuity examiners and OCT technicians do the SD-OCT scans at all visits and both will be masked to the participant study arm. The visual acuity examiners will receive the participants into the visual acuity lanes with a visual acuity case report form, study number and detail of study eye and non-study eye to be refracted, but with no previous subject records or case report forms by which the subject treatment arm could be identified. Similarly, the SD-OCT technicians will receive the subjects into the SD-OCT room on a specific case report form that provides details of subject study number and eye to be examined. The subjects will be advised at enrolment that they must not discuss the study arm they are in with the SD-OCT or Visual Acuity examiner. The retinal photographs will be graded by masked graders in the independent Reading Centre at Moorfields Eye Hospital. This will avoid performance and detection bias. We will describe the completeness of outcome data for each outcome, including reasons for attrition and exclusions from the analysis.

### Safety concerns

All adverse events and side effects will be recorded in the electronic case report form (eCRF) throughout the study regardless of their severity or relation to study participation. The light-masks are CE marked but the definitions of adverse events related to a non-CE marked device will be used to classify and report the adverse events in this study. No serious adverse events (SAEs) are expected. The PI will submit an annual report of all SAEs (expected and unexpected) to the Sponsor, and the Research Ethics Committee. The Data Monitoring and Ethics Committee (DMEC) will be provided with listings of all SAEs on an on-going basis. The study may be prematurely discontinued on the basis of new safety information, or for other reasons given by the DMEC and/or Trial Steering Committee (TSC), Sponsor, or Research Ethics Committee concerned. Following 6 months of recruitment, initial rates of recruitment will be used to project total recruitment to ensure sufficient participants to power the study. The TSC will advise on whether to continue or discontinue the study and make a recommendation to the Sponsor. If the study is prematurely discontinued, active participants will be informed and no further participant data will be collected.

### Outcomes

The primary efficacy measure is the difference between arms in the change from baseline in absolute retinal thickness at the zone of maximum thickness as determined by OCT at 24 months.

The secondary efficacy parameters at 12 and 24 months are:

Efficacy parameters:

I. Difference between arms in the change from baseline in absolute thickness at the zone of maximum thickness as determined by OCT at 12 months.
II. Other measures include:
  1. Difference between arms in the change in retinal thickness in the 9 ETDRS zones (parafoveal zones 2-5 and perifoveal zones 6-9) and macular volume.
  2. Difference between arm in morphological characteristics of macular thickness
  3. Difference between arms in the mean change in visual acuity.
  4. Difference between arms in the proportion of centre-involving macular oedema within 24 months.
  5. Difference between arms in the time to occurrence of centre-involving macular oedema.
  6. Difference between arms in the proportion requiring macular laser or antiVEGF treatment.
  7. Difference between arms in the proportion of participants that show progression of retinopathy as measured by the ETDRS severity levels and microaneurysm turnover.
  8. Compliance rates in the light mask arm.

II. Assessment of Safety Parameters {XE “6.2 Procedures for Assessing Efficacy Parameters”}

1. Difference between arms in the measures of sleep disturbance in terms of daytime sleepiness and insomnia.
2. Difference between arms in ocular and systemic adverse events and serious adverse events.

III. Assessment of Mechanistic Parameters {XE “6.2 Procedures for Assessing Efficacy Parameters”}

1. Change in P1 and N1 amplitudes and peak time in multifocal ERG after supplemental oxygen
2. Change in retinal sensitivity in scotopic microperimetry after supplemental oxygen.
3. To determine differences in change in P1 and N1 amplitudes and peak time in multifocal ERG after light-masks and dummy masks at 12 months.
4. To determine differences in change in retinal sensitivity in scotopic microperimetry after light-masks and dummy masks at 12 months.
5. To correlate the changes induced by light-masks and oxygen supplementation on retinal sensitivity using oximetry.

### Data collection

Data management procedures for the trial will be developed and overseen by King’s Clinical Trial Unit (KCTU). All baseline and follow-up data will be entered on the online InferMed MACRO electronic data capture (EDC) system (http://www.infermed.com). This system is regulatory compliant (Good Clinical Practice (GCP) and the EC Clinical Trial Directive). An eCRF using the MACRO EDC will be programmed by KCTU in collaboration with the Trial Manager, and Trial Statistician and hosted on a dedicated secure server within King’s College London. The eCRF system will have full audit trail, data discrepancy functionality, database lock functionality, and supports real time data cleaning and reporting.

The KCTU will provide training, essential documentation, and user support to the study centres, and on-site audit and monitoring. A detailed Standard Operating Procedure will cover data recording, online entry, checking, central backup and storage. A regularly updated coding manual will be developed to accompany the study database. Each authorised research worker and PI at each centre will have a unique username and password provided by the KCTU for the eCRF. The Trial Manager will provide usernames and passwords to any new researchers. Only those authorised by the Trial Manager will be able to use the system.

Baseline data will be collected and entered by researchers in each study site prior to randomisation. Each participant will be assigned a unique trial identification number at the start of the assessment process. This number will be written on all clinical assessment forms, datasheets and databases used to record participant data. Trial data will be first entered on to paper source datasheets provided to each centre during the preparation phase. The datasheets will be immediately checked for completeness and accuracy. If data queries arise, these will be logged and followed up locally before data are entered online. A hard copy of a record sheet linking patient identity, contact details and trial ID number for all participants will be kept at each site. All data will be kept secure at all times and maintained in accordance with the requirements of the Data Protection Act and archived locally according to clinical trial GCP regulations and the host institutions additional procedures.

The study incorporates a range of data management quality assurance functions. After written recording, each research worker will transcribe data onto the eCRF within one working week of a participant assessment. After completion of all follow-ups and prompt entry of data, the Trial Manager will review the data and issue queries to be answered by the research worker. At the end of the trial, the centre PI will review all the data for each participant and provide electronic sign-off to verify that all the data are complete and correct. At this point, all data will be formally locked for analysis. At the end of the trial, each centre will be supplied with a CD-ROM containing the eCRF data for their centre. This will be filed locally for any future regulatory or internal audit.

### Statistical analysis

The primary outcome will be analysed using a two-sided test from a linear mixed effect model for repeated measures across visits, which will enable a comparison between participants receiving light-masks (active) and control masks, with covariates for each follow-up visit of baseline, randomisation stratifier and arm, and with a random participant effect at each visit with unstructured covariance matrix. The primary time-point will be 24 months. The 20% allowance for dropout (trial withdrawal) is based on 18% early non-compliance observed in the pilot study and we would expect a reasonable proportion of non-compliers to provide primary time-point and intermediate visit outcome information for this analysis.

We expect the DMEC would want to monitor study power and we would regularly provide information such as non-compliance, withdrawal, and variability of the primary outcome with increasing certainty as increasing proportions of the participants pass each of the 4-monthly measurement points.

The detailed statistical analysis plan will include an additional sensitivity analysis involving all randomised participants (intention to treat strategy) examining the influence on the primary outcome analysis of opposing optimistic and pessimistic scenarios for the intervention effect in those withdrawing in each arm. We will adopt the complier average causal effects (CACE) analysis (under a missing at random assumption) as recommended and outlined by Dunn *et al*. [15].

Linear mixed effect models for repeated measures (as specified above for the primary outcome), logistic regression, and stratified Cox regression, will also be undertaken to analyse secondary and mechanistic outcomes of continuous, binary and time to occurrence type respectively. Differences will be considered significant at *P* < 0.05. Differences between the groups will be estimated with 95% confidence intervals. Repeated measure analyses (linear mixed effects models) will be used to document trends over time.

### Sample size calculation

With 300 patients (150 in each arm), we anticipate 240 to be followed up (20% dropout). This is sufficient to provide 90% power to detect 15 μm in mean change of retinal thickness at the zone of maximal thickness between arms using a 2-sided test, adjusting for baseline, at the 5% level of significance, assuming a standard deviation of 35.7 μm. The chosen detectable effect size (retinal thickness of 15 μm) is both plausible, in terms of being consistent with a confidence interval estimate for this intervention in preceding research (Ruboxistaurin trial protocol: B7A-MC-MBCU), and also minimally detectable in terms of being distinguishable from test-retest variation [25]. Detectable effect sizes for secondary outcomes based on 240 followed up (for 90% power with 5% significance level) would be a between-arm difference in mean outcome of a size that is equivalent to 0.42 of a standard deviation.

### Ethical issues

The conduct of this study will be in accordance with the recommendations for physicians involved in research on human subjects adopted by the 18th World Medical Assembly, Helsinki 1964 and later revisions. This protocol and related documents have been approved by the National Research Ethics Service Committee London - Dulwich. Local approval will be sought before recruitment may commence at the site. The Study Coordination Centre will require a written copy of local approval documentation before initiating each centre and accepting participants into the study.

The main ethical issues in relation to this study are the use of the light-masks. There are three visits that the participants need to undergo in excess of standard of care. Standard care of laser or anti-VEGF injections will be given to all those who require it. The precise risks and benefits of participating in the study will be outlined in patient information sheets, to be formulated with service user involvement.

The patients who participate in the mechanistic tests have to undergo non-invasive tests of oximetry, multifocal ERG and microperimetry. There are no known risks with these tests.

All participants will be made aware of the results of the study. If the study successfully establishes efficacy, the participants will be informed that these light-masks can be purchased but will not automatically be available in the National Health Service (NHS), though the treatment will be submitted for technology appraisal by the National Institute of Clinical Excellence (NICE) if deemed appropriate.

### Time frame

We will recruit patients over 12 months and each participant will be followed up over 24 months. The total study period is 42 months.

## Discussion

This trial is essential to confirm the role of hypoxia in the pathogenesis of DMO. The importance of publishing this extensive protocol is that it addresses a novel approach to the management of non-central DMO. If proven efficacious and safe, a light-mask is a simple non-invasive device that can help prevent the most common cause of blindness during working life that is now being managed by treatment options that have high direct and indirect costs to individuals and to the state.

Recent trials on the treatment of DMO have all focussed on centre-involving DMO and do not relate to any questions about prophylactic treatment. The prevention of any damage is obviously desirable and would lead to considerable savings in treatment costs. Additionally light-masks are simple devices, suitable for home use, and could be provided even in remote areas where there is no electricity supply. The benefit to those who cannot obtain, or pay for, present high-technology treatments is immense.

The strengths of the study are an appropriate design and sample size and to achieve measureable outcomes. The study is based on two proof-of-concept studies. The control arm will provide new information on the natural history of non-centre-involving DMO monitored using SD-OCT over 24 months. This study on light-masks is the first of its kind in the world. The limitation of the study is the inability to design a double-masked study.

## Trial status

The trial has started recruiting.

## Abbreviations

BP: blood pressure
HbA1c: glycated haemoglobin
SD-OCT: spectral domain - optical coherence tomography
mfERG: multifocal electroretinogram
ETDRS: Early Treatment of Diabetic Retinopathy Study
DMEC: DataMmanagement Ethic Committee KCTU
TSC: Trial Steering Committee
OLED: Organic Light Emitting Diode
DMO: Diabetic Macular Oedema
KHP: King’s Health Partners.
